# Potential Applications of Image-Guided Radiotherapy for Radiation Dose Escalation in Patients with Early Stage High-Risk Prostate Cancer

**DOI:** 10.3389/fonc.2015.00018

**Published:** 2015-02-02

**Authors:** Nam P. Nguyen, Rick Davis, Satya R. Bose, Suresh Dutta, Vincent Vinh-Hung, Alexander Chi, Juan Godinez, Anand Desai, William Woods, Gabor Altdorfer, Mark D’Andrea, Ulf Karlsson, Richard A. Vo, Thomas Sroka, the International Geriatric Radiotherapy Group

**Affiliations:** ^1^Department of Radiation Oncology, Howard University, Washington, DC, USA; ^2^Department of Radiation Oncology, Michael D. Wachtel Cancer Center, Oskosh, WI, USA; ^3^Department of Radiation Oncology, Medicine and Radiation Oncology PA, San Antonio, TX, USA; ^4^Department of Radiation Oncology, Martinique University Hospital, Martinique, France; ^5^Department of Radiation Oncology, University of West Virginia, Morgantown, WV, USA; ^6^Department of Radiation Oncology, Rochester Radiation Oncology Group, Rochester, NY, USA; ^7^Department of Radiation Oncology, Akron City Hospital, Akron, OH, USA; ^8^Department of Radiation Oncology, Richard A. Henson Institute, Salisbury, ML, USA; ^9^Department of Radiation Oncology, Camden Clark Cancer Center, Parkersburg, WV, USA; ^10^Department of Radiation Oncology, University Cancer Centers, Houston, TX, USA; ^11^Department of Radiation Oncology, Marshfield Clinic, Marshfield, WI, USA; ^12^University of Galveston School of Medicine, Galveston, TX, USA; ^13^Department of Radiation Oncology, Geisel School of Medicine at Dartmouth, Dartmouth College, Hanover, NH, USA

**Keywords:** prostate cancer, high-risk, image-guided radiotherapy, hypofractionation

## Abstract

Patients with early stage high-risk prostate cancer (prostate specific antigen > 20, Gleason score > 7) are at high risk of recurrence following prostate cancer irradiation. Radiation dose escalation to the prostate may improve biochemical-free survival for these patients. However, high rectal and bladder dose with conventional three-dimensional conformal radiotherapy may lead to excessive gastrointestinal and genitourinary toxicity. Image-guided radiotherapy (IGRT), by virtue of combining the steep dose gradient of intensity-modulated radiotherapy and daily pretreatment imaging, may allow for radiation dose escalation and decreased treatment morbidity. Reduced treatment time is feasible with hypo-fractionated IGRT and it may improve patient quality of life.

## Introduction

Prostate cancer is currently detected at an early stage because of routine screening for prostate specific antigen (PSA) in elderly males (1). Patients with low-risk early stage prostate cancer (PSA < 10, Gleason score < 5) demonstrate optimal results when treated with surgery or radiotherapy. However, in high-risk early stage prostate cancer patients (PSA > 20, Gleason score > 7), the current recommendation is androgen suppression therapy combined with radiotherapy because of potential for local recurrences and distant metastases (2). Increasing radiation dose to the prostate may improve local control and survival of these patients (3). However, normal organs adjacent to the prostate specifically the rectum, and bladder also receive a high radiation dose with dose escalation using the conventional three-dimensional conformal radiotherapy (3D-CRT) technique, which leads to a higher risk of complications (3). Thus, a radiotherapy technique that allows radiation dose escalation to the prostate while minimizing radiation to the rectum and bladder may improve the therapeutic ratio. Intensity-modulated radiotherapy (IMRT) has been introduced recently to decrease excessive radiation dose to the normal organs near the prostate cancer because of the steep dose gradient away from the target (4). Compared to 3D-CRT, IMRT may increase survival rates in patients with high-risk disease because of reduced rectal volume irradiated over 70 Gy and significantly decreased gastrointestinal (GI) toxicity, thus allowing radiation dose escalation to the tumor (5). However, in radiation dose escalation trials for prostate cancer, patients who had higher rectal doses during IMRT were still at an increased risk of long-term rectal complications (6). Inclusion of a large rectal volume in the IMRT planning treatment volume (PTV) may be required to avoid a marginal miss and therefore lead to excessive rectal irradiation. The introduction of image-guided radiotherapy (IGRT), which combines the normal tissue sparing effect of IMRT and daily imaging, has been proven to decrease further radiation dose to the normal organs without compromising local control in head and neck cancer (7–9). Thus, IGRT may allow for radiation dose escalation in patients with early stage high-risk prostate cancer and improve biochemical-free survival while reducing treatment toxicity.

## Techniques of Prostate Cancer Radiation Therapy Delivery with IGRT

Currently, many systems are used for daily imaging and may be classified as invasive and non-invasive. In the invasive systems, metallic markers are implanted into the prostate as fiducial markers (FM) to guide radiation delivery. Usually, three seeds are implanted under ultrasound (US) guidance into the prostate before CT planning. Daily MV or kV CT are performed to align the seeds to the planning CT before each treatment. Alternatively, the seeds can be tracked in real time imaging with a robotic system (Cyberknife) or through electromagnetic waves emitted by the transponders (Calypso System), and allow accurate radiation delivery even though the prostate moves during treatment. In the non-invasive system, the prostate position before treatment can be detected either by US or by CT scan. The patient is shifted for set-up discrepancies and additional images are obtained to verify treatment accuracy. The US system is inexpensive, simple, and non-radiation-based, but is operator-dependent. The CT system provides either kV or MV imaging. Fan beam kV CT uses a diagnostic CT scan alongside the linear accelerator. Cone beam kV CT uses a gantry mounted kV source and a flat panel detector. A series of kV X-rays are taken when the gantry rotates and a 3D image is reconstructed. Image quality is superior with fan beam kV CT compared to cone beam CT, but the couch needs to be displaced between imaging and treatment, which may lead to positioning error. In the MV CT system, the imaging is performed by the treatment beam that rotates around the patient while the couch moves (helical Tomotherapy).

Image quality is inferior with MV CT compared to kV CT, but there are no metal artifacts, which may be helpful if the patient has hip prosthesis. The latest technology for tumor imaging is based on magnetic resonance imaging (MRI), which involves a hybrid Cobalt linear accelerator and an MRI (ViewRay). This is a promising technology for prostate cancer IGRT, as MRI provides better imaging of the prostate gland compared to CT scans. Furthermore, MRI allows visualization of gross tumor volume (GTV) inside the prostate and may enable a higher radiation dose using the simultaneous integrated boost (SIB) technique.

## Reduction of Prostate Cancer Treatment Morbidity Associated with Accurate Image Guidance

Preliminary evidence suggests that better visualization of the prostate during radiotherapy leads to improved patient quality of life (QOL) because of the increased accuracy of radiation delivery. In a study of 282 prostate cancer patients treated with 3D-CRT with (*n* = 154) and without image guidance (*n* = 128), proctitis severity was significantly reduced with image guidance (10). Patients who had image guidance underwent prostate FM placement and MRI imaging before radiotherapy planning to outline the target volume. Rectal and urinary dysfunction during radiotherapy was assessed with QOL questionnaires. Despite a higher tumor dose to the prostate, patients treated with image guidance experienced less diarrhea and rectal pain compared to the ones who did not undergo IGRT. During radiotherapy, the prostate position changes daily depending on bladder and rectal filling. Accurate target localizing with intra-prostate FM instead of bony landmark fusion leads to a decreased volume of bladder and rectum being irradiated to a high dose and therefore a reduction of acute morbidity. Gill et al. (11) reported significant reduction in severe urinary frequency, diarrhea, and fatigue in patients with prostate cancer who were treated with IGRT (*n* = 265) compared with patients not treated with IGRT (*n* = 26). Both groups were treated with IMRT using the same constraints and PTV margins. The prostate dose was higher for the group treated with IGRT (78 Gy) compared to those treated without IGRT (74 Gy). Thus, regardless of the radiotherapy technique, accurate localization of the prostate before treatment decreases treatment toxicity by eliminating the geographic miss of the prostate, which would lead to an excessive radiation dose to the adjacent non-involved normal structures. Other studies have corroborated the image guidance effects on sparing normal organs in patients with prostate cancer (12, 13).

## Radiation Dose Escalation for High-Risk Prostate Cancer Patients Treated with IGRT and Conventional Fractionation

In patients with high-risk prostate cancer, long-term follow-up suggests that a radiation dose ≥80–81 Gy to the tumor bed may be required for long-term biochemical control (14, 15). The effect of radiation dose escalation may be independent of hormonal therapy (15). Radiation doses up to 86.4 Gy were determined to be feasible with limited toxicity in prostate cancer patients treated with IMRT (16). In a study of 1,002 prostate cancer patients treated to 86.4 Gy with IMRT, late grade 3 GI and genitourinary (GU) toxicities were reported (0.7 and 2.2%, respectively). However, acute grade 3–4 toxicities were not reported in the study. Thus, IGRT may allow for radiation dose escalation and further reduction of normal tissue toxicity by combining IMRT and daily imaging. Indeed, preliminary evidence suggests that IGRT as a safe radiotherapy technique for radiation dose escalation in patients with prostate cancer. Takeda et al. (17) reported no acute grade 3–4 toxicities in 141 patients with intermediate or high-risk localized prostate cancer when the radiation dose was increased from 76 Gy (*n* = 13) to 80 Gy (*n* = 128). Only two patients developed long-term grade 3 toxicities. Kok et al. (18) compared late toxicities among 311 patients treated with IMRT for prostate cancer without image guidance (74 Gy) and with image guidance (78 Gy). Despite a higher radiation dose in this study, late GI toxicities were significantly reduced in patients with image guidance. In a similar study comparing IGRT (78 Gy) and 3D-CRT (76 Gy) for high-risk prostate cancer, late GI and GU toxicities were significantly decreased for patients treated with IGRT (19).

## Radiation Dose Escalation for High-Risk Prostate Cancer Patients Treated with IGRT and Hypofractionation

Prostate cancer cells are characterized by a low α/β ratio ranging from 0.8 to 2.2 Gy, suggesting that delivering a radiation dose higher than the conventional fractionation of 1.8–2 Gy/day may be more effective for cancer cell killing. On the opposite side is the risk of normal tissue injury associated with high dose hypofractionation. However, if the volume of rectal and bladder tissue exposed to a high radiation dose can be reduced with high precision radiation delivery, hypofractionation may be an ideal radiation technique to reduce treatment time while potentially improving the biochemical control in patients with high-risk prostate cancer. Jerekzek-Fossa et al. (20) compared the acute toxicity of 179 prostate cancer patients treated with IGRT [70.2 Gy in 2.7 Gy/fraction (84.2 Gy dose equivalent in 2 Gy/fraction)] and 174 patients treated with 3D-CRT (80 Gy in 2 Gy fraction). There was no significant difference in toxicity between the two groups of patients. In a follow-up study, QOL of the patients treated with hypo-fractionated IGRT was not significantly affected long-term, thus illustrating that IGRT may be beneficial in patients with high-risk prostate cancer (21). A similar radiation dose escalation study was performed in 48 prostate cancer patients using regimens of 68.04 Gy at 2.52 Gy/fraction (*n* = 32), 70 Gy at 2.5 Gy/fraction (*n* = 5), and 70.2 Gy at 2.6 Gy/fraction. No patients developed grade 3–4 late toxicities (22). The safety of IGRT for hypofractionation was also corroborated in another study where patients with high-risk prostate cancer were treated up to 74.2 Gy in 2.65 Gy/fraction with the SIB technique. Only 1 out of 70 patients developed an acute grade 3 rectal reaction (23). A preliminary report from a randomized study comparing high dose hypofractionation to conventional fractionation with IGRT suggests that hypo-fractionation may allow for a higher radiobiologic dose without increasing treatment toxicity for high-risk prostate cancer patients. Patients (*n* = 124) were recruited and treated to 76 Gy in 2 Gy/fraction (*n* = 57) and 63 Gy in 3.15 Gy/fraction (*n* = 67) equivalent to 84 Gy in 2 Gy/fraction to the prostate with the SIB technique (24). There was no significant difference in acute toxicities between the two arms suggesting that IGRT may confer effective normal tissue sparing but long-term follow-up is needed as complications may develop later.

The extreme hypo-fractionation scheme for high-risk prostate cancer involves continuous tracking of the prostate with two orthogonal X-ray imagers that allow a tight PTV margin posteriorly (3 mm) while multiple non-coplanar beams improve the plan conformity compared to IMRT (Cyberknife) (25). Total treatment time can be reduced to 1 week instead of the conventional 8–9 weeks of treatment as the volume of rectum and bladder exposed to high radiation dose can be minimized. In addition, if treatment toxicity can be reduced, a high dose to the prostate may be feasible to improve local control. Oliai et al. (26) reported the acute toxicity and long-term complications of 70 patients with low- to high-risk prostate cancer treated with radiation dose escalation by Cyberknife (CK) ranging from 35 Gy in 7 Gy/fraction (*n* = 5), 36.25 Gy in 7.25 Gy/fraction (*n* = 36), and 37.5 Gy in 7.5 Gy/fraction (*n* = 29). Acute and late grade 3 GU toxicities were 4 and 3%, respectively. None of the patients experienced grade 3–4 toxicities at a median follow-up of 31 months. Katz et al. (27) also corroborated the low toxicity of CK for prostate cancer. Among 304 patients with low (*n* = 211), intermediate (*n* = 81), and high-risk (*n* = 12) prostate cancer treated with CK to 35 Gy in 7 Gy/fraction (*n* = 50) and 36.25 Gy in 7.25 Gy/fraction (*n* = 254), none of the patients developed acute grade 3–4 toxicity. At a median follow-up of 60 months, only 2% of the patients developed long-term grade 3 GU toxicity. A follow-up study suggested that prostate cancer patients treated with this fractionation on CK had QOL similar to that observed in conventional fractionation (28). Even though most patients treated with CK hypo-fractionation had low to intermediate risk prostate cancer, late grade 3 GI and GU toxicities ranged from 0 to 3%, which confirmed the safety of CK for radiation dose escalation (29–34). The high radiobiologic equivalent dose of 92 Gy (corresponding to 35 Gy in 7 Gy/fraction with an α/β ratio of 1.5) or higher with hypo-fractionated CK suggests that this radiotherapy technique may be potentially effective for high-risk prostate cancer. However, this hypothesis will need to be tested in future prospective trials. It is encouraging that in a pooled analysis of patients with prostate cancer treated with hypo-fractionated CK, a 5-year PSA relapse-free survival of 81% was suggested for high-risk patients.

## Role of Brachytherapy in Radiation Dose Escalation in Patients with High-Risk Prostate Cancer

Brachytherapy may be the ideal modality of radiation dose escalation for prostate cancer either alone or as a boost. The radioactive seeds or high dose rate (HDR) brachytherapy catheters are inserted inside the prostate, thus prostate motion is not an issue for brachytherapy compared to external beam radiation. In addition, the radiation dose decreases proportionally to the square of the distance away from the radioactive sources and allows significant sparing of the rectum and bladder. Because of the risk of extra-capsular extension of the tumor, brachytherapy is frequently given as a boost following external beam radiation, but brachytherapy without external beam radiation has been reported with excellent local control and survival in selected studies when combined with androgen deprivation therapy (35–37). A higher dose to the prostate ranging from 10,000 to 12,000 cGy may be achieved as a boost after external beam radiation with low dose rate (LDR) brachytherapy (38, 39). As a result, long-term biochemical-free survival has been observed with LDR brachytherapy boost for high-risk prostate cancer (40). Brachytherapy with HDR begins to play a prominent role in the management of high-risk prostate cancer because of the technical issues associated with LDR permanent seeds implant: discrepancy between planned and actual seeds distribution, inability to correct seeds position or to optimize the dose delivered once the seeds are in place, which may be related to the radiation oncologist technical skills. The HDR catheters are relatively easy to visualize with US and may be safely implanted outside the prostate capsule and into the seminal vesicles without the risk of seeds migration. Uncertainty over target dose associated with prostate volume changes, which occurs following LDR seeds implant is not an issue with HDR brachytherapy. Perhaps the most important advantage of HDR over LDR brachytherapy is the real time dose modulation, which provides immediate feedback to the physician and physicist for optimal catheter distribution and dwell time. Preliminary results of HDR monotherapy or combined with external beam radiotherapy were very encouraging with excellent biochemical-free survival and acceptable toxicity (35–37, 41, 42). Dose escalation with HDR boost after external beam radiotherapy was feasible and was reported to be associated with a higher biochemical-free survival in high-risk prostate cancer patients (43, 44). As most studies reported 3D-CRT with HDR brachytherapy, it would be interesting to combine IGRT and HDR brachytherapy for high-risk prostate cancer to decrease long-term complications in future prospective studies. Nevertheless, brachytherapy is an invasive procedure with its complications and may not be indicated for all patients. The optimal radiation dose for disease control in high-risk prostate cancer has not been elucidated when combined with androgen deprivation therapy and needs to be investigated in future clinical trials.

## Current Controversies in the Management of High-Risk Prostate Cancer

### Role of pelvic radiotherapy in the treatment of high risk of prostate cancer

As high-risk prostate cancer patients may have occult pelvic lymph nodes metastases that are not detected with current diagnostic technologies, pelvic lymph nodes irradiation may improve loco-regional control and survival. However, these patients also received androgen deprivation therapy that may affect micrometastases. On the other hand, treating the prostate and seminal vesicles only allows radiation dose escalation without the toxicity of pelvic irradiation. Radiation dose escalation from 64 to 74 Gy to a local field with 3D-CRT and neoadjuvant androgen deprivation has been reported to improve biochemical-free survival in a randomized study (45). Using the Roach formula to estimate the risk of pelvic lymph nodes metastases, a matched-pair analysis did not report the benefit of adding pelvic radiotherapy in patients at high risk (>15%) of lymph nodes metastases (46). Two randomized studies also corroborated the lack of survival benefit when the pelvis was radiated compared to irradiation of the prostatic bed only (47, 48). As an illustration, excellent local control and survival were observed in studies where HDR brachytherapy to the prostate were combined with hormonal therapy without pelvic irradiation (35–37, 49). The rate of distant metastases or pelvic failures did not increase in those studies. Thus, pelvic irradiation may not be necessary for high-risk prostate cancer when combined with androgen deprivation therapy and may increase treatment toxicity because of the increased volume of normal tissues in the radiation fields.

### Prostate motion and optimal PTV for IGRT

Prostate motion depends on rectal and bladder filling. In the supine position for treatment, prostate position is less affected by bladder filling because of bladder extension anteriorly. However, in the prone position, pressure on the bladder may push the prostate posteriorly and the prostate position is affected by the patient breathing pattern (50). Prostate motion ranged from 1.5 to 3.7 mm, 0.7 to 1.9 mm, and 1.4 to 3.6 mm in the antero-posterior (AP), left-to-right (LR), and superior–inferior (SI) dimension, respectively (51–55). The magnitude of prostate motion also depends on the individual patient. Choice of a PTV also depends on whether the institution includes pelvic lymph node irradiation or not as it would be difficult to tract the motion of two independent systems. In that sense, radiation dose escalation would be technically easier without pelvic irradiation. PTV margins for the lymph nodes may need to be enlarged to avoid under dosing the pelvic lymph nodes if the set up needs to change because of prostate motion secondary to rectal filling. The choice of the PTV margins also depends on the prostate tracking system used by the institution, either with FM or electromagnetic transponders (invasive method), or with soft tissue fusion based on CBCT or MVCT. For the non-invasive method, once an optimum PTV has been selected by the institution, adaptive therapy performed for the first week or two of treatment based on observed prostate motion on an individual patient may allow the clinician to reduce the PTV for example if the established PTV may be too generous. These efforts may reduce interfraction motion but will not reduce intrafraction motion that occurs during treatment. Unless the institution uses an online tracking system for treatment delivery such as the robotic CK, PTV margins should take into account intrafraction motion, which may be dependent on treatment time. The use of an endorectal balloon may decrease intrafraction prostate motion and may reduce further rectal dose by pushing the posterior rectal wall away from the high dose area (56). Another factor to take into consideration is the deformation of the prostate, which may be related to peristalsis, degree of pelvic musculature contraction, and breathing pattern. Any prospective trial on radiation dose escalation should take into consideration the degree prostate deformation during treatment as under dosing of the prostate may occur in patients with a large degree of prostate deformation (more than 10% of prostate volume) (57).

In summary, PTV margins should be determined by the type of IGRT image tracking and radiation treatment delivery. For example, published recommended LR, AP, and SI margins ranged from 3.6, 3.7, and 3.7 mm and 2.46, 2.28, and 2.56 mm for FM and CBCT, respectively (58, 59).

### Dose prescription comparison among institutions using prostate IGRT

Currently, there is no standard recommendation on how to prescribe radiation dose for patients undergoing prostate IGRT. Each institution set up a specific protocol making dose comparison difficult among various institutions to assess long-term local control, and complications. In addition, there were different dose schedule fractionation, and various PTV margins based the technology used for image tracking. As an illustration, Norkus et al. (24) reported the following protocol in a randomized study of dose escalation using hypofractionation and CBCT for image guidance. The prostate PTV was treated to a total dose of 76 Gy at 2 Gy/fraction and 63 Gy at 3.15 Gy/fraction, respectively, with a PTV margin of 10 mm except posteriory (7 mm). The dose was optimized so that 95–108% of the PTV received the prescribed dose. On the other hand, Takeda et al. (17) treated the prostate PTV to a total dose of 80 Gy in 2 Gy/fraction using FM with a PTV margin of 5 mm except posteriorly (3 mm). The dose was prescribed to cover 95% of the target volume. At another institution, even though FM was used for image guidance, the prostate PTV margin was 10 mm except posteriorly (6 mm). The prescribed dose was 86.4 Gy to a maximum isodose encompassing the PTV (60). As the preliminary results from these institutions are excellent for local control with acceptable toxicity, it would be very difficult to set up a standard recommendation for PTV margins and dose.

## Future Directions of Image-Guided Radiotherapy for Radiation Dose Escalation in Patients with High-Risk Prostate Cancer

Patient with high-risk prostate cancer often have a heterogeneous tumor distribution within the prostate with areas of concentrated cancer cells responsible for disease recurrence following radiotherapy (61). These intra-prostatic tumor nodules may require a higher dose to achieve local control. The conventional radiotherapy technique with IMRT or IGRT for prostate cancer consists of a homogeneous radiation dose distribution within the prostate. Thus, previous studies of radiation dose escalation increased the total dose to the prostate, which may account for the risk of late GI and GU complications. New imaging techniques such as diffusion-weighted (DW), MRI, magnetic resonance spectroscopy (MRS), and positron emission tomography (PET)-CT are more accurate for detection of these intra-prostatic tumor nodules and may allow for radiation dose escalation within the prostate (62–64). Dosimetric studies suggest that radiation dose escalation for intra-prostatic tumors nodules with IGRT is feasible and may allow significant reduction of the rectal dose (65). In a clinical study of 118 patients with prostate cancer, the GTV defined by MRI or MRS was treated to 80–81 Gy while the prostate dose was limited to 78 Gy with IMRT. No patients developed grade 3–4 GI toxicity (66). Thus, IGRT may be a promising technique for radiation dose escalation on intra-prostatic nodules as significant rectal toxicity has been observed in patients who had hypo-fractionated IGRT up to 50 Gy in 10 Gy/fraction (67).

## Conclusion

Image-guided radiotherapy is a promising technique to reduce treatment toxicity in patients with early stage high-risk prostate carcinoma and may improve biochemical-free survival associated with radiation dose escalation. Reduced treatment time with hypo-fractionated IGRT may improve patient QOL as they will have more time to spend with their family. Future clinical trials focused on improving tumor imaging with MRI, MRS, and PET-CT to reduce long-term complications associated with high dose prostate irradiation are warranted.

## Conflict of Interest Statement

The authors declare that the research was conducted in the absence of any commercial or financial relationships that could be construed as a potential conflict of interest.
